# Gender-Specific Effects of Cognitive Load on Social Discounting

**DOI:** 10.1371/journal.pone.0165289

**Published:** 2016-10-27

**Authors:** Tina Strombach, Zsofia Margittai, Barbara Gorczyca, Tobias Kalenscher

**Affiliations:** Comparative Psychology, Heinrich Heine University Düsseldorf, Universitätsstraße 1, 40225, Düsseldorf, Germany; Radboud Universiteit, NETHERLANDS

## Abstract

We live busy, social lives, and meeting the challenges of our complex environments puts strain on our cognitive systems. However, cognitive resources are limited. It is unclear how cognitive load affects social decision making. Previous findings on the effects of cognitive load on other-regarding preferences have been ambiguous, allowing no coherent opinion whether cognitive load increases, decreases or does not affect prosocial considerations. Here, we suggest that social distance between individuals modulates whether generosity towards a recipient increases or decreases under cognitive load conditions. Participants played a financial social discounting task with several recipients at variable social distance levels. In this task, they could choose between generous alternatives, yielding medium financial rewards for the participant and recipient at variable social distances, or between a selfish alternative, yielding larger rewards for the participant alone. We show that the social discount function of male participants was significantly flattened under high cognitive load conditions, suggesting they distinguished less between socially close and socially distant recipients. Unexpectedly, the cognitive-load effect on social discounting was gender-specific: while social discounting was strongly dependent on cognitive load in men, women were nearly unaffected by cognitive load manipulations. We suggest that cognitive load leads men, but not women to simplify the decision problem by neglecting the social distance information. We consider our study a good starting point for further experiments exploring the role of gender in prosocial choice.

## Introduction

In today’s societies, we have to accomplish a multitude of different tasks in parallel. We are on the phone with a business partner while simultaneously scanning through the headlines of the newspaper, drinking our coffee and keeping upcoming duties in mind–all at the same time. In this state of mind, we have to make decisions that might affect our own future, but very often, our choices also impact other people in our social environment. The question arising is how we deal with the strain of cognitive load when making social decisions that potentially affect others, given that our cognitive resources have limited capacity [1], to decide and act efficiently. And how does our behavior change when the cognitive capacity is exceeded? Here, we aim to investigate the role of cognitive load on social decision making [2,3].

Most studies on the effects of limited cognitive resources on behavior investigate decision making in an isolated social environment. For instance, it has been shown that cognitive load manipulations alter learning [1], aviation [4] and user interface design [5]. However, in real life, many, if not most of our decisions involve considering the well-being of others. But, unfortunately, the role of cognitive load in social decisions is little understood, with previous research showing ambiguous results on the role of cognitive load on social preferences [6–15]: while some studies on prosocial choice behavior in which subjects make decisions affecting the payoff of other participants report that subjects became more generous towards their interaction partner under cognitive load conditions [9,11], others demonstrated increased selfishness [13–16]. Additionally, many publications do not report any effect of cognitive load on social decision making [6–8,17]. Next to procedural differences, the social choice designs used in these studies differed in the degree of familiarity, or social closeness, between the participants and their interaction partners. For example, in some studies, the interaction partner was anonymous, whereas in others, he/she was introduced to the participants, and yet in other studies, the interaction partners were actual acquaintances or co-students [18]. Interestingly, whether the interaction partner was anonymous or not appeared to determine the sign of the cognitive load effects on generosity: when the interaction partner was an anonymous stranger, subjects often became more generous under cognitive load conditions [9,11], but when the interaction partner was familiar to the subject, e.g. the interaction partner was a co-student or introduced before, he/she became more selfish [16,18]. When familiarity was not well controlled, any putative effects of cognitive load on social preferences might have been obscured by the uncontrolled variability in anonymity. We therefore hypothesize that the ambiguity in previous results might be evoked by differences in the degree of social distance between participants and their interaction partners. This hypothesis blends in with recent theories on prosociality and cognitive control. These theories suggest that prosocial behavior requires self-control to resolve the conflict between selfish and other-regarding motives [2,12,18,19]. Because evidence suggests that self-control capacities become exhausted with increasing cognitive load ([20–22], but see [23]), putting strain on the cognitive control system is therefore likely to change social-distance-dependent generosity profiles.

In the present study, we investigate the effect of cognitive load on prosocial decision making with socially close, socially distant and unknown interaction partners. We systematically vary social distance using a social discounting paradigm [2,3,24]. Social discounting refers to the idea that generosity towards others diminishes systematically over social distance between donor and recipient, with social distance indicating how much, or how little, the donor cares about the recipient. In this context, we hypothesize that cognitive load affects prosocial, other-regarding decision making and we further propose that this effect is modulated by the social distance between donor and recipient. More specifically, we expect that, under high cognitive load conditions, individuals become less generous towards people at closer social distance, but more generous towards people at large social distance, thus showing less variability in generosity across social distance. We use a psychometric approach to address this question. In a financially incentivized social discounting task, participants indicated their level of generosity towards recipients at variable social distances. We fitted a hyperbolic social discount function [2,3,24] to our participants’ choice data to mathematically capture their social discounting behavior. We expected that cognitive load flattened the social discount function, reflecting the hypothesized social-distance-dependent cognitive-load effects on generosity. Finally, since one recent study identified different psychological predictors of social discounting in men and women [25], we also suggest a potential gender difference in cognitive load effects on social discounting.

The present study has important implications for theories of social decision making, but will also inform research on business settings. Insights into the effect of cognitive load on social preferences might lead to a better understanding why high workload, stress and other states characterized by cognitive preoccupation often also result in interpersonal distress. Moreover, a multitasking environment is common in the business world. Understanding the impact of cognitive load on decision making and other-regarding behavior might help to create working environments that are more productive and less prone to exhaustion, occupational stress and work-related depression.

## Materials and Methods

### Participants

88 Participants (34 male, M_age_ = 23.09, SD_age_ = 2.69, Range_age_: 18–30) were recruited at the University of Düsseldorf. The participants were randomly assigned to either the control condition (low cognitive load; N = 44, 17 men), or the experimental condition (high cognitive load; N = 43, 17 men). Participants who had previously participated in psychological experiments as well as students enrolled in Psychology or Economics were excluded from participation. One subject stopped the experiment during the procedure and was excluded from the analysis. Written consent was obtained before the experiment started. The study was approved by the local ethics committee and conformed to the guidelines of the Declaration of Helsinki. Participants were financially compensated for their participation, as outlined below. The study was fully incentive-compatible, did not involve deception and thus met the standards in psychology and behavioral economic research.

### General experimental procedure

Participants were randomly assigned to a high (experimental) and low (control) cognitive load manipulation. Before the start of the main experiment, a brief self-control scale was administered (see below for details). After completing the scale, participants received all instructions and information about the procedure. Subsequently, they were subjected to the cognitive load manipulation (high versus low cognitive load) and then performed the social discounting task (see below for details on the cognitive load manipulation and social discounting task). They were debriefed after they finished the experiment.

### Cognitive load manipulation

Cognitive load was elicited using two different treatments. Both treatments were similar to previous tasks used in studies on self-regulation and ego-depletion [21,26,27]. For the stimulus-detection task, all participants received typewritten sheets of paper with a text extracted from an advanced machine learning book [28]. In the control condition, participants were asked to cross off every *e* they found in the text. In the experimental condition, instructions were similar, but participants received an extra set of rules as follows: they were instructed to cross off every *e*, except if the *e* was followed by a vowel and except if the *e* was the beginning letter of a word. However, when the word that began with an *e* was at the beginning of the sentence, the *e* had to be crossed off. In an unstructured interview after the procedure, participants in the experimental group indicated more often than participants in the control group that the procedure was exhausting.

In addition to the instructions, the control and the experimental conditions also differed with regard to font size and font transparency of the text (Control: font size: 14, brightness: 0%, experimental condition: font size: 9, brightness: 75%). This made it more difficult and effortful for the participants in the experimental condition to read the text. Both groups had five minutes to work on the task.

The stimulus-detection task was followed by a computer-based Stroop task [29–32], programmed in Presentation (Neurobehavioral Systems). The Stroop task is a thoroughly validated tool to induce cognitive load [29,32]. Subjects in the experimental group saw a color word displayed with differently colored fonts on a computer screen (six different colors). They were asked to indicate, by pressing a corresponding button on a keyboard, either the semantic meaning of the displayed word, or the name of the color of the font used to display the word, respectively. The meaning of the presented word was sometimes incongruent with the color of the font used. For example, the word ‘blue’ may have been presented in green fonts. Congruent (font color and semantic meaning are identical) and incongruent (font color and semantic meaning are dissimilar) trials were presented in 12 blocks (subjects had to indicate the words’ semantic meanings in six blocks, and their font color name in six other blocks) with 24 trials in random order. In order to perform this task, subjects in the experimental condition had to suppress the automatism to read the semantic meaning of the word. In the high cognitive load condition, incongruent trials were presented in 50% of the trials. The incongruence between font-color and word-meaning was not present in the control group, where subjects were always asked to indicate the semantic meaning of the color-word, independent of the font-color used. To simplify the task even more, the words were always presented in grey fonts to avoid incongruences. Performance in the control condition therefore required less suppression of the automatism to read out the word instead of indicating the color. In both groups, words were presented on a white screen. Inter-stimulus intervals had a duration of 500ms in which a fixation cross was presented. Maximum response time was limited to 5000ms.

After the stimulus detection and the Stroop tasks were completed, the experiment continued with the social discounting task [2,3].

### Social discounting task

Social discounting was measured with the same paradigm used in our previous studies on social discounting [2,3], (see [3] for a discussion of the elicitation procedure of social distance). To introduce the concept of social distance, each participant was shown a scale consisting of 101 icons, with the leftmost icon representing the participant and the others representing his social environment. Participants were told that social distance 1 (the most leftward icon closest to the participant) represents the socially closest person, while distance 100 (the most rightward icon) would be a stranger who they may have randomly met on the street. Social distance 50 stands for a distant acquaintance, whose name they may not know. Once participants were familiar with the concept of social distance, they were asked to write down the names of representatives for the following social distances: 1, 2, 3, 5, 10, 20. Although distances 50 and 100 were also included in the experiment, participants were not required to provide a name as these distance levels often represent remote individuals. Participants were specifically asked not to include anyone in their list against whom they have negative feelings.

**Fig 1:**
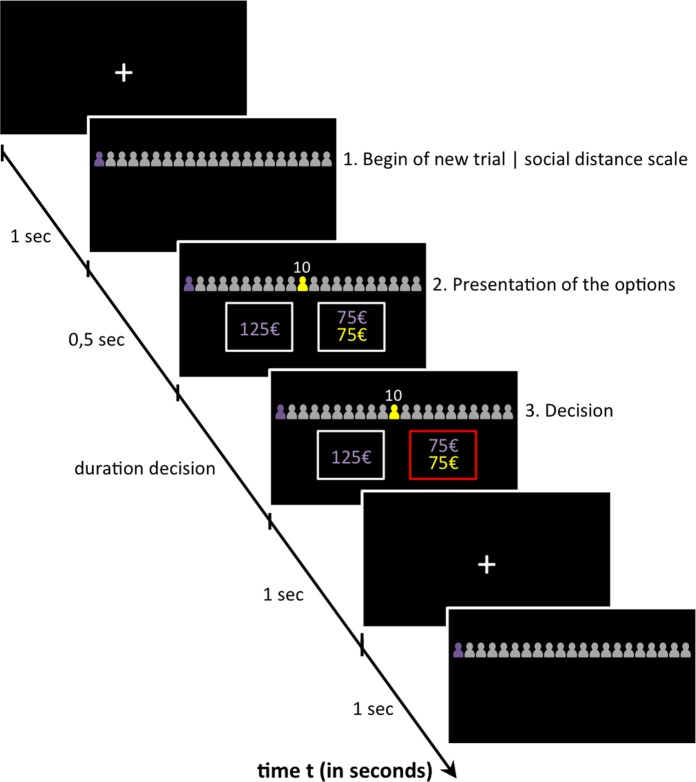
An example trial in the social discounting task. Social distance information was given on top of the screen, the options below. The subject could choose between a selfish reward just for herself or a generous option, yielding a reward for herself and another person at the indicated social distance. The side of presentation of the options was randomized. As soon as both options were presented, the participant could make her decision. The participant’s choice was fed back by a red box around the chosen option. Note that this figure has been adjusted for illustration purposes; stimulus size and screen format are not to scale with the presentation dimensions used testing. In addition, the figure displays only 21 icons, instead of 101 icons shown during scanning, to facilitate perceptibility.

In each trial a yellow icon on the social distance scale indicated the social distance of the recipient. To avoid perceptual issues with the visual representation of social distance the social distance information was additionally indicated by a number on top of the yellow icon (cf. Fig 1). Participants had to choose between a selfish option, yielding a large reward for themselves, and a generous option, yielding a smaller reward for them and the same amount for the recipient at the indicated social distance. The selfish reward varied between €75 and €165, with increments of €10. The generous option was identical in all trials, yielding €75 for the participant and €75 for the recipient on the specific social distance. For example, in a given trial, a subject may choose between a €125 reward only for herself (selfish option), or a €75 reward for herself and a €75 reward for a recipient on social distance 20 (generous option). In total, the participants made 160 decisions– 8 social distances, 10 selfish rewards and all combinations were presented twice. The order of trials as well as the side of the presentation of the selfish and generous choice alternatives was fully randomized (cf. Fig 1).

Participants were informed during the instructions before the experiment that, at the end of the task one of their decisions would be randomly chosen and 10% of its payoff would be paid out, therefore they and potentially another person would be able to earn money based on their decisions. The money the participant allocated to herself was paid out directly after the experiment. For generous decisions, subjects were asked to indicate the address of the other person in the randomly chosen trial. If the randomly chosen trial was about an anonymous person or stranger, e.g. at higher social distances, a random person on the campus of the University of Düsseldorf, Germany received the reward.

### Brief self-control scale

Before the experimental procedure started, self-control was measured with a German translation of the Brief Self-Control Scale (BSCS; 33; available at www.uni-konstanz.de/diagnostik/research_measures.htm). The BSCS consists of 13 items indicating agreement with given statements on a 5-point-Likert scale to quantify a subjective measure of self-control. The scale was intended as a control for potential inhomogeneous group differences.

### Data analysis

The analysis procedure was identical to procedures used previously [2,3,24]. First, we determined for each subject and each social distance the point at which a subject was indifferent between the selfish and the generous option. To this end, logistic regression was used to identify the point at which the probability of answering generously or selfishly was 50%. For each indifference point, we calculated the individual amount forgone, i.e. the difference between own-reward of the selfish alternative and own-reward of the generous alternative. For example, if a participant was indifferent between receiving €125 just for herself and €75 for herself and €75 for a recipient at a specific social distance she was willing to forego €50 to increase the wealth of the recipient by €75. The individual amount forgone at a given social distance level measures how much it was worth to the participant to endow the recipient with €75; it can therefore be construed as a social premium a subject was willing to pay to improve the recipient’s wealth. This social premium served as a social-distance-dependent estimate of the other-regarding value a subject attaches to increasing a recipient’s wealth by €75.

If the participant made exclusively selfish or generous choices at a given social distance, indifference points were determined to be €170 or €70, respectively (for more details on the estimation of the discount curve see [3]). After determining the individual amounts forgone for each social distance played, the following standard hyperbolic model was fitted to the social-distance-dependent social premiums [2,3,24]:

$$v=\frac{V}{\left(1+kD\right)}$$ (1)

where *V* symbolizes the magnitude of a reward received by a recipient at social distance *D*. The parameter *v* refers to the amount forgone, i.e. the social premium a subject is willing to pay in exchange for endowing the recipient on social distance *D* with €75. Thus, *v* can be interpreted as a proxy of the socially discounted other-regarding value of improving the wealth of another individual at social distance *D*. *V* is the intercept with the y-axis and determines the height of the social discount function. Thus, *V* can be interpreted as the level of generosity towards socially close recipients. The degree of discounting is described by the parameter *k*, which indicates the steepness and shape of the hyperbolic discount curve.

## Results

The goal of the current experiment was to identify the effect of cognitive load on social discounting, i.e. on social distance-dependent generosity. We hypothesized that a higher cognitive load alters the social discount function. More specifically, we expected flatter social discounting after high cognitive load manipulations.

### Cognitive Load Manipulation

There was no significant difference in the self-control scores of the BSCS between participants of the control and the experimental conditions (BSCS: M_control_ = 3.13, SD_control_ = 0.60; M_CognitveLoad_ = 3.17, SD_Cognitive Load_ = 0.59; t-test: t(85) = 0.282, p = 0.779, η^2^ = 0.001).

As manipulation check for the cognitive load manipulation, we compared the reaction times in the Stroop task between experimental and control group. We assumed that higher cognitive load would go along with longer reaction times [11]. We found a significant difference between experimental and control subjects in reaction times (M_control_ = 792.50 ms, SD_control_ = 132.78; M_CognitveLoad_ = 862.71 ms, SD_Cognitive Load_ = 155.00; t(85) = -2.271; p = 0.026, η^2^ = 0.057) and errors made in the Stroop task (M_control_ = 4.34, SD_control_ = 5.26; M_CognitveLoad_ = 10.79, SD_Cognitive Load_ = 8.94; t(68) = 4.087; p<0.001, η^2^ = 0.164), supporting our assumption that cognitive load was higher in the experimental Stroop condition compared to the control condition. We additionally tested whether performance in the Stroop task differed between men and women. However, a mixed 2x2 ANOVA with the factors group and gender revealed no significant main effect of gender, or interaction effects between gender and condition, for reaction times (all p>0.40). For the e-crossing task we checked whether subjects in the experimental condition made less progress in identifying “*e”*s compared to subjects in the control condition because of differences in task difficulty and perceptibility of the text. To this end, we counted the letters that were processed by the participant until time-out. One participant failed to complete the task, and was thus excluded from all further analyses. As for the remaining participants, we found a significant difference in the total number of letters processed between experimental and control subjects (M _cognitive load_ = 1478.36, SD _cognitive load_ = 466.37; M _control_ = 2071.18, SD _control_ = 436.40; t(84) = 6.09; p < 0.001, η^2^ = 0.306). Again, there was no indication of gender main and interaction effects on performance in the e-crossing task (all p>0.20). Thus, in line with others [21,33,34] we assumed that the differences in complexity of the e-crossing task between experimental and control groups translated into differences in cognitive load.

### Social Discounting

One subject failed to complete the social discounting task, and was thus excluded from analyses. For each remaining subject, we fitted the hyperbolic social discount function (Eq 1) to the individual amounts forgone, i.e., the social premiums. We used the best-fitting discount parameters *V* and log(*k)* to quantify and compare social discounting between experimental and control groups. *k* was log transformed to approximate a normal distribution. Occasionally, the hyperbolic function could not be fitted to a participant’s data, e.g., because he invariantly selected the same alternative. In those cases, the respective log(*k*)-values were replaced by the corresponding group averages. As stated earlier, *V* can be interpreted as the level of generosity towards socially close recipients, and *k*, or log(*k*) respectively, indicates the steepness of the curve, thus how steeply generosity decays across social distance. In both conditions, generosity levels, measured as the amount forgone at indifference points (the social premiums, see methods), decreased across social distance, replicating previous studies on social discounting [2,3,24,35].

**Fig 2:**
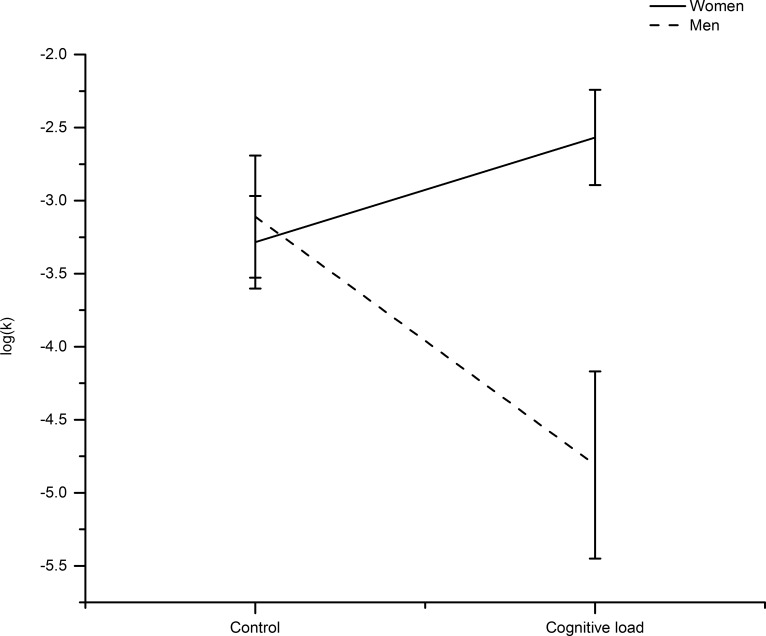
Mean log(k) values in the experimental groups. While men’s performance was significantly affected by the cognitive load manipulation, women’s performance did not differ between the two experimental conditions. Error bars indicate +/- 1SEM.

We hypothesized that cognitive load flattens the social discount function, reflecting the predicted decrease in generosity towards socially close recipients, and increase in generosity towards socially more distant people. To test this hypothesis, we first compared the log-transformed *k*-values between the two experimental conditions. Contrary to our prediction, a t-test did not indicate a significant difference in log(*k*)-values between the groups (M _control_ = -3.22, SD _control_ = 1.66; M _cognitive load_ = -3.48, SD _cognitive load_ = 2.35; t(73) = 0.586, p = 0.560, η^2^ = 0.004). As indicated earlier, it has been suggested that men and women differ in their social preferences [25]. This implies that social discounting may be driven by distinct, gender-dependent motives. Thus, to further inspect our data, we ran additional analyses including gender as an additional factor in a 2x2 analysis of variance (ANOVA) with cognitive load and gender as fixed factors. The ANOVA revealed a significant interaction effect between cognitive load and gender on the log(*k*)-values (F(82,1) = 8.375, p = 0.005, η_p_^2^ = 0.093; Fig 2). These results seem to corroborate our hypothesis that cognitive load affects social preferences differently in men and women.

**Fig 3:**
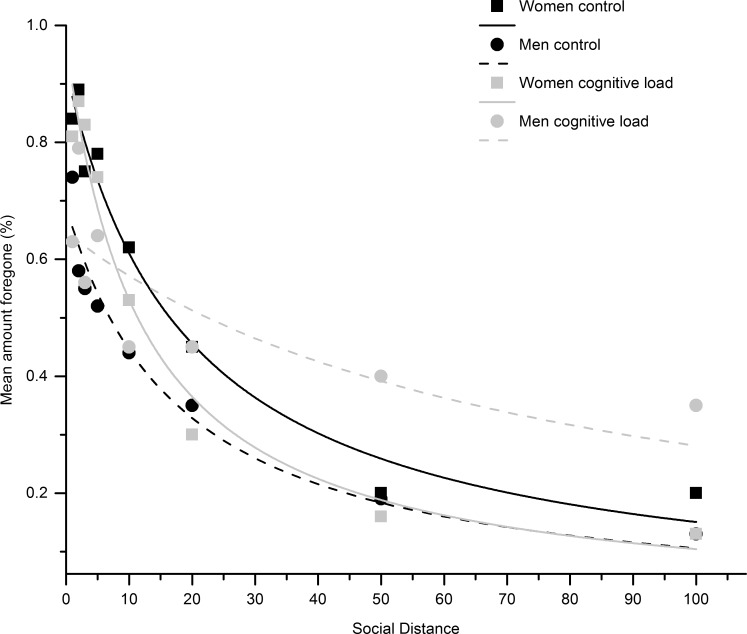
A standard hyperbolic model was fitted to the amounts forgone (social premiums). Social discount curves are presented separately for gender (men; women) and high and low cognitive load. Compared to men, women seemed to be overall more generous towards socially close others. While there was no significant difference in the overall social-distance-dependent decline in generosity in women between high and low cognitive load conditions, men under high cognitive load were less sensitive towards variations in social distance, reflected by a considerably flatter discount curve.

To further characterize the gender x cognitive load interaction, post-hoc analyses revealed that cognitive load effects on log(*k*) were most pronounced in men: while men had lower log(*k*)-values under high- compared to low-cognitive-load conditions (M _men & control_ = -3.11, SD _men & control_ = 1.72, M _men & cognitive load_ = -4.81, SD _men & cognitive load_ = 2.64; t(32) = 2.22, p = 0.033, η^2^ = 0.133), women did not show significant differences in log(*k)*-values between the cognitive load treatments (M _women & control_ = -3.28, SD _women & control_ = 1.65, M _women & cognitive load_ = -2.57, SD _women & cognitive load_ = 1.63, t(50) = 1.57, p > 0.1). Furthermore, compared to log(*k*)-values of women, men’s log(*k*)-values were significantly lower in the high cognitive load condition (t(40) = 3.404, p = 0.002, η^2^ = 0.23; see Fig 2). Thus, while social discounting behavior in women seemed relatively unaffected by cognitive load, men showed flatter social discounting under high cognitive load (see Fig 3).

Next, we repeated the fixed factor 2x2 ANOVA to investigate the effects of gender and cognitive load on the second parameter in the hyperbolic function *V*. While cognitive load did not seem to affect *V*, the analysis revealed a significant effect of gender, indicating that women (M = 1.08, SD = 0.32), on average were overall more generous to close others than men (M = 0.87, SD = 0.38) irrespective of cognitive load (main effect of gender: F(82, 1) = 7.325, p = 0.008, η_p_^2^ = 0.082, main effect of cognitive load: F(82,1) = 0.13, p = 0.719, η_p_^2^ = 0.002, cognitive load x gender interaction: F(82,1) = 0.502, p = 0.481, η_p_^2^ = 0.006, see Fig 2).

In sum, our data suggest that cognitive load affected the steepness, but not necessarily the height, of the social discount function in a gender-specific way. However, there might be several alternative accounts for the interaction effect of cognitive load and gender on log(*k*) [36,37]. For instance, it is possible that men, unlike women, simply made more noisy decisions under high- than under low-cognitive load conditions. To rule out this possibility, we performed additional analyses to establish putative effects of cognitive load on decision noise as follows: as outlined earlier, we used binary logistic regression to determine the individual indifference points between generous and selfish options for each social distance level. Decision noise as well as high choice variability and/or inconsistent decision patterns should be reflected in poorer goodness-of-fit measures of the individual logistic regressions. We therefore used the individual goodness-of-fit estimates R^2^ as a measure of decision noise and choice inconsistency. In cases subjects always and invariantly selected the selfish over the generous option, or vice versa, R^2^ was set to one. To test whether men differed from women in the number of noisy or inconsistent decisions under high compared to low cognitive load, we calculated the mean adjusted R^2^ across all indifference points for each subject, and used it as the dependent variable in a 2x2 ANOVA with gender (men/women) and level of cognitive load (control/experimental condition) as fixed factors. The ANOVA revealed no significant main or interaction effects of gender and cognitive load on adjusted R^2^-values (all p > 0.1). We thus have no evidence to assume that the above reported gender-specific cognitive-load-effects on social discounting can be explained by cognitive-load-dependent decision noise and/or choice inconsistency.

Another potential explanation for the low log(*k*)-values in men in the high cognitive load condition might be differential attention-effects: after high cognitive challenge, men may simply pay less attention to the task and just “click” through the experiment, randomly choosing any option, or perseverating on one response option. Subjects had to indicate their selection of the generous or selfish option by clicking on the left or right option presentation (cf. Fig 1). Since the side of the presentation of the options was randomized, perseveration on one side, or random clicking, would result, for each social distance level, in a medium indifference point estimate that would be similar for all social distances. This may explain the lower log(k) values in men in the high cognitive load condition: because of the fact that indifference point estimates would be similar across social distance, fitting a hyperbolic function to the choice data obtained from a perseverating or random “clicker” would yield flatter discounting, and consequently lower log(*k*)-values. We assumed that such mindless, random or perseverating “clicking”-behavior should be reflected by reduced reaction times [38]. To address the possibility that men became mindless “clickers” under high cognitive load conditions, we calculated mean reaction times per individual and ran another 2x2 ANOVA with gender and cognitive load as independent factors and mean reaction time as dependent variable. Again, we did not find any significant main or interaction effects (all p > 0.1). Thus, mindless random or perseverating clicking behavior is not likely to be the reason for above reported gender-specific cognitive-load-effects on social discounting.

Finally, we checked for correlations between the discount parameters log(*k*), *V*, the number of errors and reaction times in the Stroop task. None of the correlations reached significance (all p > 0.1).

## Discussion

We investigated the effect of cognitive load on social discounting. We exposed participants to either a high or a low cognitive load manipulation in which they had to perform two tasks requiring cognitive control (the e-crossing and the Stroop task). They subsequently performed a social discounting task in which they repeatedly decided between a large reward for themselves (the selfish option), or a smaller reward for them plus an additional reward for recipients at variable social distances (the generous option). We replicated previous findings on social discounting showing that a generosity metric–the willingness to forego a reward in exchange for increasing the wealth of the recipient (the social premium)—decreased over social distance. In line with our prediction, we found that high cognitive load flattened the social discount function, but this cognitive-load effect on social discounting was only found in men. Following the cognitive load manipulation, men showed considerably flatter social discounting than women as well as men in the control condition, indicating that, after increased cognitive load, the typical decrease in generosity across social distance was much less pronounced. By contrast, the discount function of female subjects was similar in the high- and low-cognitive-load conditions. Furthermore, we found that cognitive load only exerted its effect on the overall decline in generosity across social distance. For the closest social distance, measured by the parameter *V* of the social discount function, generosity was gender-dependent, with men being less generous than women, but remained unaffected by cognitive load.

Our finding that cognitive load affects social discounting in a gender-specific way blends in with recent dual process models of decision making [11,39–43]. These dual process models postulate that decisions are based on the interplay between two complementary mental processes: while affective processes deal with emotional and automatic behavioral responses, cognitive processes are responsible for controlled, deliberated behavior. One important difference between the two processes is that controlled processes have a limited capacity [11], but see [23,41]. Thus, when self-control or cognitive effort is exerted, the cognitive processes eventually get exhausted and automatic processes might gain stronger influence on decision making. Previous research indicates that there are a multitude of factors, besides gender, influencing whether automated or cognitive processes are used [10,11,17,39,40,42,44]. Amongst others, mood, low blood glucose levels, exhaustion of willpower and cognitive load determine whether the cognitive or affective processes have a greater impact on decision making [11,44–46]. The dual process approach thus states that, when cognitive capacity is high, decisions can be made in a more controlled way and presumably more in line with long-term interests by integrating different aspects into the decision making process. However, when the cognitive resources are exhausted, the automatic processes dominate. Thus, the amount of available cognitive control seems to shape which preferences are revealed in a decision.

Recent theories on altruism and other-regarding preferences suggest that prosocial behavior requires cognitive control to overcome selfish motives [2,19]. Research evidence also suggests, that women exhibit higher self-control than men [47]. Combining these insights with the predictions of the dual process model, we speculate that the results in our present study reflect a gender-dependent switch from controlled to automatic processing: while men use more automatic processes to make social decisions when cognitive load is high, women consistently rely on deliberate control processing independent of cognitive load. The disuse of the deliberative system in men is likely to lead to a reduction in the information complexity that is processed to form a decision [43]. In order to come up with a sound decision in the social discounting task, participants have to consider own- and other-reward magnitudes as well as social distance to the recipient. One way to simplify the decision problem is to reduce the dimensionality of the social choice alternatives. It is therefore possible that men under high cognitive load conditions neglected the social distance dimension of the choice problem. Because women were presumably less sensitive to the cognitive load manipulation, and more sensitive to the contextual parameters of the decision making task [48] the switch from deliberative to automatic processes was less pronounced, and women consequently showed no difference in social discounting behavior between cognitive load conditions. However, since research findings on gender specific effects of cognitive load on decision making are scarce, additional studies are necessary to corroborate the present findings. We consider our study a good starting point for further experiments.

In addition to the gender specific effect of cognitive load on the overall decline in generosity across social distance, we also found a difference between males and females at the extreme close end of the social distance scale measured by the V parameter of the social discount function. More specifically, males were less generous than females towards the person they felt closest to. As the person at social distance 1 is almost always a family member, or next of kin, this finding fits well with previous research indicating that women are overall more likely to help family members and next of kin than men [49–51]. Interestingly, in contrast to the effect of cognitive load on men’s decline in generosity across social distance, prosocial behavior towards the person at social distance 1 remained unaffected by cognitive load in men. This can be explained by the fact that helping kin does not require self-control [2,52].In summary, we suggest that our findings that cognitive load affects social discounting in a gender-dependent way might be interpreted within the dual process framework [11,40,41]. We propose that cognitive load diminishes deliberation capacities in men and, thus, the likelihood of activating the controlled, cognitive system. As such, decisions taken under additional cognitive load are governed to a greater extent by automatic choice heuristics. This might lead to a reduction in the choice alternatives’ dimensions that are considered to form a decision [53,54]. In the present case, we propose that the social distance dimension is considered less under increased cognitive load. In line with previous research, we suggest that the cognitive-load-induced diminution of deliberation capacities is more pronounced in men than women. Our data are interesting for a couple of reasons. First, we show that cognitive-load effects on prosocial sentiments are complex and dependent on several interacting factors, including social distance and gender. Since social distance and gender were often not, or only partially controlled in previous studies on cognitive load and social choice, our results may help reconcile the inconsistencies in earlier findings. Second, previous studies are incongruous regarding gender difference in cognitive load effects on cognition. We show that gender effects only become visible in prosocial choice tasks when social distance is taken into consideration. Thus, only the combination of social distance, gender and a cognitive load manipulation is able to uncover the effect of cognitive load on social behavior. In sum, we suggest that inclusion of social distance in social experiments might be advantageous.

Our results highlight the importance of research on the effects of cognitive load on social behavior. Insights in this field might lead to a better understanding of behaviors in situations where cognitive capacity is scarce, i.e. in jobs that demand multitasking faculties, or executive decisions that are made under time pressure and stress. A better understanding could help to develop strategies to deal with the risks of cognitive exhaustion to improve the quality of decisions. That might also lead to optimize the work environment to improve the quality of the decisions made in a work-related setting. Finally, we suggest that social preferences as well as social distance should be included in economic models and psychological theories to further their descriptive and predictive value.

## Conclusions

The effects of cognitive load on generosity and prosocial behavior are complex, and depend on social distance between donor and recipient, as well as the gender of the donor.

The gender-difference in the impact of cognitive load on social behavior suggests that men and women process social information differently when their cognitive capacities are burdened with high cognitive load. Our findings have implications for our understanding how a person’s environment might influence her or his ability to make decisions.
